# Concentrations and pharmacokinetic parameters of MRI and SPECT hepatobiliary agents in rat liver compartments

**DOI:** 10.1186/s41747-021-00236-y

**Published:** 2021-09-21

**Authors:** Catherine M. Pastor, Florian Joly, Valérie Vilgrain, Philippe Millet

**Affiliations:** 1 Centre de recherche sur l’inflammation, Inserm, U1149, CNRS, ERL8252, Université de Paris, F-75006 Paris, France; 2 Department of Radiology, University Hospital of Geneva, Rue Gabrielle-Perret-Gentil, 4, 1205 Geneva, Switzerland; 3 Department of Radiology, Hôpital Beaujon, Hôpitaux Paris Nord Val de Seine (AP-HP), 92110 Clichy, France; 4 Department of Psychiatry, University Hospital of Geneva, Geneva, Switzerland; 5 Department of Psychiatry, Faculty of Medicine, University of Geneva, Geneva, Switzerland

**Keywords:** Membrane transport proteins, Cholestasis, Hepatocytes, Bile canaliculi, Extracellular space

## Abstract

**Background:**

In hepatobiliary imaging, systems detect the total amount of agents originating from extracellular space, bile canaliculi, and hepatocytes. They add *in situ* concentration of each compartment corrected by its respective volume ratio to provide liver concentrations. *In vivo* contribution of each compartment to liver concentration is inaccessible. Our aim was to quantify the compartmental distribution of two hepatobiliary agents in an *ex vivo* model and determine how their liver extraction ratios and cholestasis (livers lacking canalicular transporters) might modify it.

**Methods:**

We perfused labelled gadobenate dimeglumine (Bopta, 200 μM, 7% liver extraction ratio) and mebrofenin (Meb, 64 μM, 94% liver extraction ratio) in normal (*n* = 18) and cholestatic (*n* = 6) rat livers. We quantified liver concentrations with a gamma counter placed over livers. Concentrations in hepatocytes and bile canaliculi were calculated. Mann-Whitney and Kruskal-Wallis tests were used.

**Results:**

Hepatocyte concentrations were 2,043 ± 333 μM (Meb) *versus* 360 ± 69 μM (Bopta, *p* < 0.001). Meb extracellular concentrations did not contribute to liver concentrations (1.3 ± 0.3%). The contribution of Bopta extracellular concentration was 12.4 ± 1.9% (*p* < 0.001 *versus* Meb). Contribution of canaliculi was similar for both agents (16%). Cholestatic livers had no Bopta in canaliculi but their hepatocyte concentrations increased in comparison to normal livers.

**Conclusion:**

Hepatocyte concentrations are correlated to liver extraction ratios of hepatobiliary agents. When Bopta is not present in canaliculi of cholestatic livers, hepatocyte concentrations increase in comparison to normal livers. This new understanding extends the interpretation of clinical liver images.

## Key points

- The *ex vivo* model quantifies the compartmental distribution of gadobenate dimeglumine and mebrofenin.
- Hepatocyte concentrations are correlated to the liver extraction ratio of hepatobiliary agents.
- Contribution of bile canaliculi to liver concentrations is similar for gadobenate dimeglumine and mebrofenin (16%).
- Cholestatic livers had low gadobenate dimeglumine concentrations in canaliculi but hepatocyte concentrations increased in comparison to normal livers.

## Background

The quantification of liver function in cirrhotic patients is important to evaluate the tolerance to partial hepatectomy and estimate prognosis. Besides various plasma biomarkers, imaging with hepatobiliary agents is another option to quantify liver volume and function [1–4]. The function is quantified by imaging parameters such as the maximum liver enhancement or activity, the time to obtain the maximal signal, and the elimination half-life of agents from livers [5–7].

Several hepatobiliary agents such as gadobenate dimeglumine (Bopta), gadoxetic acid, and mebrofenin (Meb) are transported inside hepatocytes across the sinusoidal Organic anion transporting polypeptide (Oatp). To efflux from hepatocytes, agents cross the Multidrug resistance associated protein 2 (Mrp2) located on the canalicular membrane and Mrp3 on the sinusoidal membrane. Hepatocyte concentrations reflect the balance between these uptake and excretory transporter activities. When Oatp disappears in advanced hepatocellular carcinoma, the tumour appears hypointense on hepatobiliary phase in comparison to surrounding hepatocytes that retain sinusoidal transporters [8–10]. Besides hepatocytes, imaging also relies on concentrations inside the extracellular compartment and bile canaliculi. However, this compartmental distribution (or contribution of each compartment to liver concentrations) is inaccessible *in vivo*.

Hepatobiliary imaging detects the total amount of agents originating from the extracellular space, bile canaliculi, and hepatocytes. The system adds the *in situ* concentrations of each compartment corrected by its respective volume ratio to liver to provide liver concentrations [11]. Thus, in cirrhotic livers, parenchymal hypointensity on hepatobiliary phase reflects the increased volume ratio of the extracellular space when fibrosis replaces hepatocytes. The hepatocyte volume decreases and *in situ* hepatocyte concentrations are impaired by the low expression of Oatp [12, 13]. Volume ratio of bile canaliculi to liver and *in situ* concentrations are unknown in cirrhosis.

The isolated and perfused rat liver is an *ex vivo* model of pharmacokinetic research which can quantify the compartmental distribution of transporter-dependent hepatobiliary imaging [14]. Hepatobiliary agent concentrations are measured in hepatic veins and common bile duct. By placing a gamma counter over livers, it is possible to mimic an imaging system and quantify liver concentrations during the perfusion of Bopta or Meb. A pre-perfusion of gadopentetate dimeglumine (Dtpa) quantifies the extracellular concentrations, whilst concentrations in bile canaliculi are calculated from bile concentrations and the volume ratio of canaliculi to liver [15].

The aim of our study was to quantify the compartmental distribution of two hepatobiliary agents in an *ex vivo* model and determine how their liver extraction ratios and cholestasis (livers lacking canalicular transporters) might modify it. From the accumulation and decay profiles of hepatocyte concentrations, new pharmacokinetic parameters will be described to better understand hepatobiliary images.

## Methods

### Isolated and perfused rat livers

A total of 24 livers were evaluated. Livers from 18 normal male Sprague-Dawley rats were isolated and perfused under anaesthesia (Pentobarbital, 50 mg kg^−1^, i.p.). The protocol was carried out in accordance with the Swiss Guidelines. It was accepted by the institutional ethical Committee (University of Geneva) and approved at the veterinary office in Geneva (No. 1006/3384). We also perfused Bopta in six cholestatic rats lacking Mrp2 [16].

The abdominal cavity was opened, and the portal vein was cannulated [17]. The abdominal vena cava was transected and an oxygenated Krebs-Henseleit-bicarbonate (KHB) solution was pumped into the portal vein, the solution being discarded by a vena cava transection. The liver flow rate (*Q*_H_) was slowly increased up to 30 mL/min. A cannula was inserted in the right atrium and the abdominal inferior vena cava ligated, allowing solutions perfused through the portal vein to efflux by hepatic veins.

**Fig. 1:**
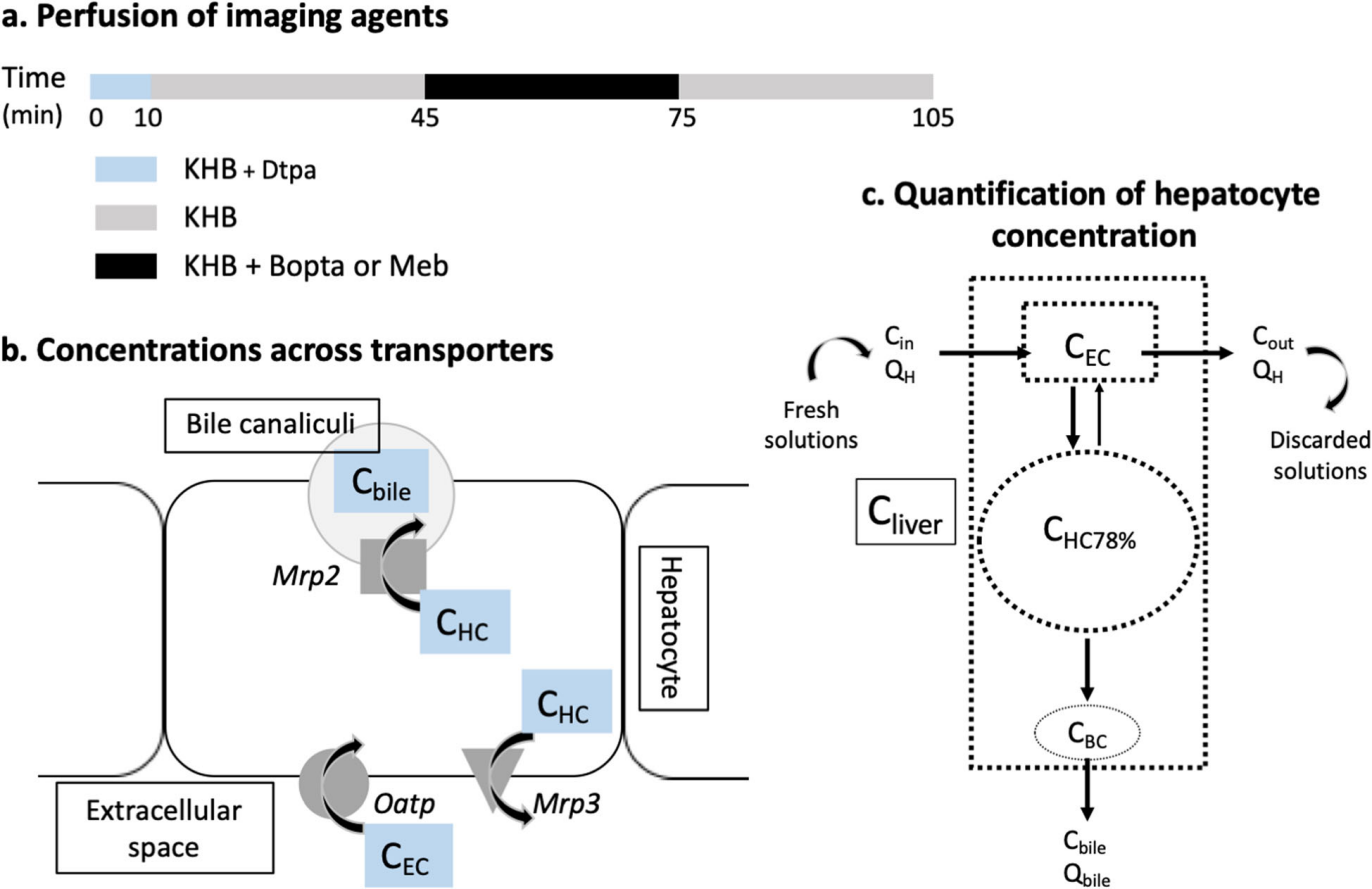
**a** Perfusion of imaging agents. Livers (*n* = 15) were successively perfused with Krebs-Henseleit bicarbonate solution (KHB) + 200 μM ^153^Gd-Dtpa, KHB, KHB + 200 μM ^153^Gd-Bopta, and KHB. Nine additional livers were perfused with KHB + 64 μM ^99m^Tc-Dtpa, KHB, KHB + 64 μM ^99m^Tc-Meb, and KHB. Illustration of Bopta and Meb transport across Oatp, Mrp2, and Mrp3 (**b**) and quantification of hepatocyte concentration (**c**). Extracellular concentration (*C*_EC_) is quantified during Dtpa perfusion. *C*_HC78%_ for hepatocyte concentration measured by the counter. *In situ* concentration in bile canaliculi is similar to concentration in common bile duct (*C*_bile_). Bile canaliculi concentration measured by the counter (*C*_BC_) is *C*_bile_ · 0.0043 (volume ratio of canaliculi to liver). *C*_HC78%_ is defined by *C*_liver_ − *C*_EC_ − *C*_BC_. Liver flow rate (*Q*_H_), bile excretion rate (*Q*_bile_), concentration in portal vein (*C*_in_), and concentration in hepatic veins (*C*_out_)

Livers were perfused with KHB solution ± imaging agents using a nonrecirculating system: livers were perfused with fresh solutions and solutions flowing from hepatic veins were discarded (Fig. 1c). The common bile duct was cannulated to collect bile samples and measure bile concentration (*C*_bile_, μM) and bile flow rate (*Q*_bile_, μL/min/liver) every 5 min. Agent concentrations in hepatic veins (*C*_out_, μM) were collected every 5 min.

We diluted agents in the KHB solution that contain 118 mM NaCl, 1.2 mM MgSO_4_, 1.2 mM KH_2_PO_4_, 4.7 mM KCl, 26 mM NaHCO_3_, and 2.5 mM CaCl_2_. The solution was equilibrated with a mixture of 95% O_2_–5% CO_2_. The electrolytic composition and pH were normal. We did not add proteins in the solution and agent entry into livers was not modified by protein binding within sinusoids. Unknown protein binding in extracellular space, hepatocytes, and bile canaliculi might modify Bopta and Meb pharmacokinetics. However, Bopta and Meb have a low protein binding that should not interfere with transfer rates across compartments [18, 19].

### Perfusion of imaging agents (Fig. 1a)

Fifteen livers (nine normal and six cholestatic livers lacking Mrp2) were perfused with gadopentetate dimeglumine (Dtpa; Magnevist®; Bayer imaging) to measure the liver extracellular concentrations because the agent distributes only into this compartment. After a rinse period to eliminate Dtpa from livers (within 5 min), livers were perfused with the hepatobiliary agent gadobenate dimeglumine (Bopta, MultiHance®; Bracco Imaging). Bopta distributes in the extracellular space, hepatocytes, and bile canaliculi. Both agents were labelled with ^153^Gd by adding ^153^GdCl_3_ (1 MBq/mL) to commercially available (0.5 M) solutions used for patients. ^153^Gd-Dtpa and ^153^Gd-Bopta were diluted to a 200-μM concentration. Bopta perfusion was followed by a rinse period with KHB solution.

Nine normal livers were perfused with Technescan Dtpa® (Dtpa, b.e.imaging GmbH) and mebrofenin (Meb; Choletec®; Bracco Imaging). Dtpa and Meb (25 mg) were labelled with the same radiotracer ^99m^Tc (7 and 11 MBq, respectively). Both solutions are commercially available for patients. Dtpa distributes only in the extracellular compartment whilst Meb distributes in the extracellular space, hepatocytes, and bile canaliculi. Both agents were diluted to a 64-μM concentration. Dtpa and Meb perfusion were followed by a rinse period with KHB solution. The protocol period lasted 105 min.

To quantify agent concentrations in liver compartments, a gamma counter that collects count rates every 20 s was placed 1 cm above a right liver lobe. At the end of experiment, Bopta or Meb liver amounts were measured by an activimetre and related to the last count rates. Bopta or Meb concentrations in the common bile duct and hepatic veins were measured with a gamma counter. Concentrations in large vessels, bile ducts, and livers are expressed in μM units. We considered that 1 g of liver was close to 1 mL.

### Calculation of hepatocyte concentrations (Fig. 1c)

The gamma counter delineated a region of interest from which Bopta or Meb amounts originating from the extracellular, bile canaliculi, and hepatocyte compartments were divided by the rat liver weight to obtain liver concentrations (*C*_liver_, μM). Concentrations in the extracellular compartment (*C*_EC_, μM) were constant during the perfusion of Dtpa. The concentrations detected by the counter were similar to the *in situ C*_EC_ because the distribution volume is 1. We assumed that concentrations inside the bile canaliculi were similar to those measured in the common bile duct (*C*_bile_, μM). The concentrations of bile canaliculi detected by the counter (*C*_BC_) were *C*_bile_ · 0.0043 according to Blouin et al. [20] who measured the volume ratio of bile canaliculi to liver in rat biopsies. The hepatocyte concentrations (*C*_HC78%_) detected by the counter were equal to *C*_liver_ − *C*_EC_ − *C*_BC_ and originate from 78% of the liver volume according to Blouin et al. [20]. To convert *C*_HC78%_ measured by the counter into *in situ* concentration into hepatocytes (*C*_HC100%_), we applied the equation: *C*_HC100%_ = (100/78) × *C*_HC78%_.

### Profiles of liver concentrations over time

To analyse Bopta or Meb liver accumulation over time, we searched for nonlinear regressions that fit the data using the GraphPad Prism software (version 8, GraphPad Software, La Jolla CA, USA). In normal livers, data fitted either a continuous hinge function (segmental regression lines with gentle connexion) [21] or a segmental linear regression with abrupt connexion [22]. Both regressions were described by two lines *L*_1_ and *L*_2_ intersecting at *T*_0_ (min) or time when bile excretion starts to impact the accumulation. Before *T*_0_, the slope of *L*_1_ (_slope_*L*_1,liver_ in μM/min) characterises Bopta or Meb uptake into hepatocytes across Oatp. After *T*_0_, the slope of *L*_2_ (_slope_*L*_2,liver_ in μM/min) characterises the balance between entry and efflux transporter activities.

During the rinse period, no agent enters into liver and concentration decay fitted a one-phase decay *Y* = (*Y*_0_ − plateau) · *e*^−*kel* · X^ + plateau. The software provided a decay (elimination) rate constant (*k*_el,liver_ in min^−1^) and a half-time (*T*_1/2,liver_ in min) or time to reach 50% of the initial concentrations. With the assumption that the hepatocyte volume (*V*_HC_, mL) was 78% of liver weight, the elimination clearance from hepatocytes (CL_el,HC_ in mL/min) was calculated by CL_el,HC_ = *k*_el,HC_ × *V*_HC_.

Liver concentrations were acquired every 20 s by the counter but this acquisition frequency is difficult to implement in clinical imaging. Therefore, we simulated decreased acquisition frequencies (2, 5, and 10 min) and determined the lower frequency that gives *T*_0_, _slope_*L*_1,liver_, _slope_*L*_2,liver_, and *k*_el,liver_ similar to those obtained at a 20-s acquisition frequency.

### Profiles of hepatocyte concentrations over time

Liver pharmacokinetic parameters do not fully characterise the transporter functions of hepatocytes because liver concentrations also include extracellular and canaliculi concentrations. Consequently, we measured all parameters using hepatocyte concentrations and compared them to those obtained in livers.

### Statistical analysis

Data are given as means ± SD. To compare Bopta or Meb parameters and to determine the influence of Mrp2 absence on Bopta parameters, we used a Mann-Whitney test (GraphPad Prism version 8, GraphPad Software, San Diego, USA). To compare the effect of acquisition frequencies on pharmacokinetic parameters, we used a Kruskal-Wallis test with multiple comparisons between frequencies. Difference between groups was considered significant for *p* < 0.05.

## Results

### Compartmental distribution of Bopta and Meb concentrations

**Fig. 2:**
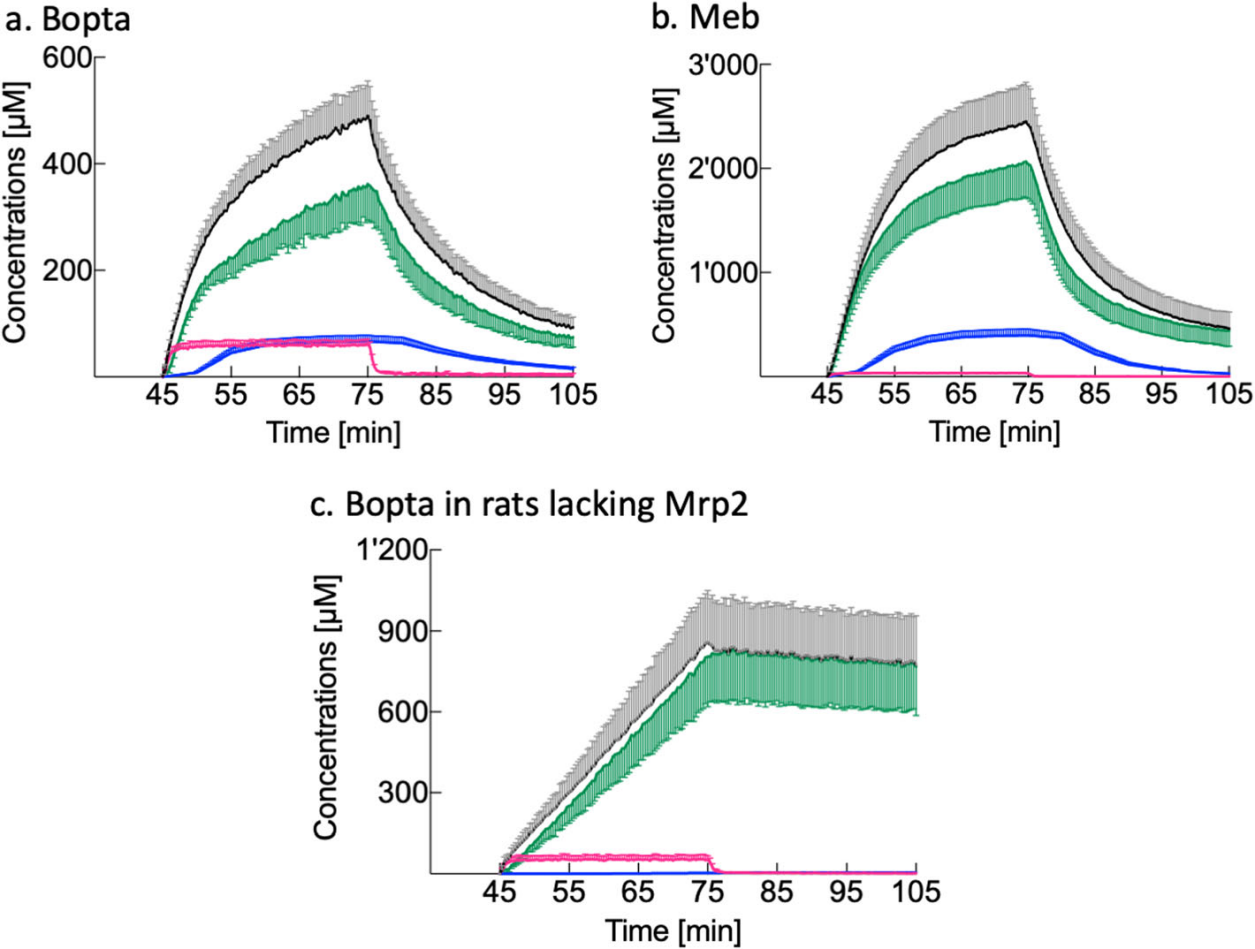
Bopta and Meb compartmental distribution. **a** Normal livers (*n* = 9) perfused with Krebs-Henseleit bicarbonate solution (KHB) + 200 μM Bopta (45–75 min) and KHB (75–105 min). **b** Normal livers (*n* = 9) perfused with KHB + 64 μM Meb (45–75 min) and KHB (75–105 min). **c** Cholestatic livers lacking Mrp2 (*n* = 6) perfused with KHB + 200 μM Bopta (45–75 min) and KHB (75–105 min). Liver concentrations (black symbols) are measured by the counter. Concentrations in extracellular compartment (red symbols) are measured during the previous Dtpa perfusion (data not shown). Concentrations that originate from bile canaliculi (blue symbols) and from 78% hepatocytes (green symbols) are calculated

Figure 2 and Table 1 describe how liver extraction ratios and cholestasis interfere with the compartmental distribution of hepatobiliary agents. Meb has a 94% liver extraction ratio and provided much higher maximal *C*_HC78%_ (2,043 ± 333 μM) than Bopta (360 ± 69 μM, *p* < 0.0001) which has a 7% liver extraction ratio. Consequently, Meb *C*_out_ in hepatic veins was low, whilst Bopta *C*_out_ was only slightly lower than the perfused concentrations (Table 1). Meb concentrations in the extracellular space did not contribute much to liver concentrations (Table 1, 1.3 ± 1.9%). The maximal Meb concentrations detected by the counter in bile canaliculi (*C*_BC_) were 393 ± 61 μM and accounted for 17 ± 4% of liver concentrations. In contrast, maximal Bopta *C*_EC_ (60 ± 10 μM) participated to liver concentrations all along the accumulation period (12.4 ± 1.9%, *p* < 0.001 *versus* Meb). Maximal Bopta *C*_BC_ was 66 ± 11 μM and accounted for 14 ± 4% (no difference with Meb, *p* = 0.09). The lack of Mrp2 in cholestatic livers induced low Bopta *C*_BC_, increased Bopta accumulation in hepatocytes, and Bopta hepatocyte trapping during the decay period (Fig. 2c and Table 1).

**Table 1:** Bopta and Meb concentrations (end of accumulation period)

Imaging agents	Bopta	Meb	*p*, Bopta ***versus*** Meb	Bopta
Livers	Normal	Normal		no Mrp2
*C*_in_ [μM]	200	64		200
*C*_out_ [μM]	185 ± 2	5 ± 2	< 0.001	186 ± 3^ns^
*C*_EC_ [μM]	60 ± 10	31 ± 7	< 0.001	52 ± 16^ns^
*C*_BC_ [μM]	66 ± 11	393 ± 61	< 0.001	3 ± 1**
*C*_HC78%_ [μM]	360 ± 69	2,043 ± 333	< 0.001	804 ± 185**
*C*_HC100%_ [μM]	462 ± 88	2,619 ± 427	< 0.001	1,031 ± 237**
*C*_bile_ [μM]	15,734 ± 2,380	94,529 ± 9,759	< 0.001	713 ± 171**

Statistics between normal livers and livers lacking Mrp2 perfused with Bopta (column 2 *versus* column 5) are nonsignificant (ns) and *p* < 0.001 (**). *C*_*EC*_ Concentration in extracellular space, *C*_*BC*_ Concentration in bile canaliculi detected by the counter, *C*_*bile*_ *In situ* concentration in bile canaliculi and common bile duct, *C*_*HC78%*_ Concentration in hepatocytes detected by the counter, *C*_*HC100%*_ *In situ* hepatocyte concentrations

### Accumulation and decay profiles of Bopta and Meb liver concentrations

**Fig. 3:**
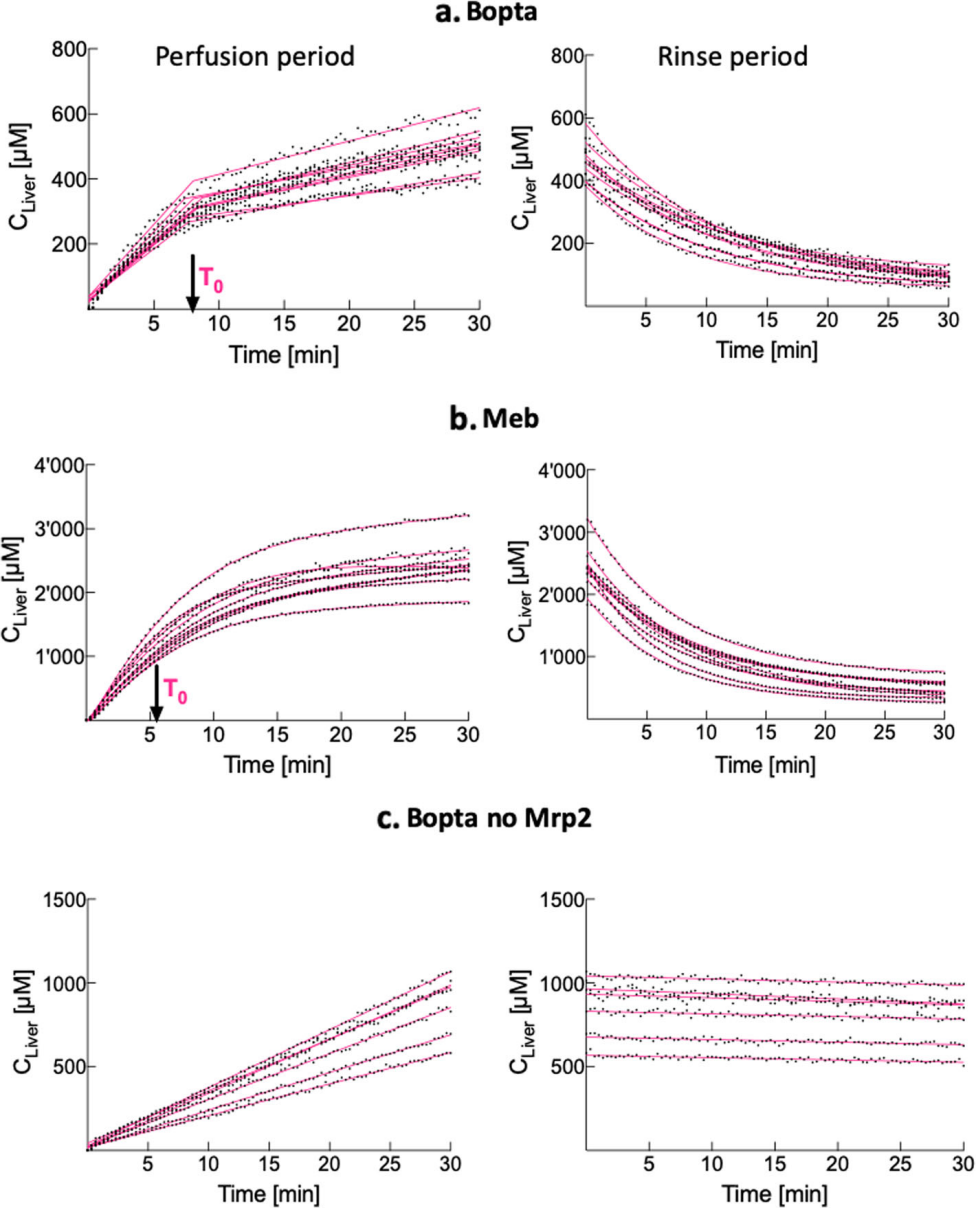
Accumulation and decay of Bopta and Meb liver concentrations. **a** Normal livers (*n* = 9) perfused with Krebs-Henseleit bicarbonate solution (KHB) + 200 μM Bopta (left panel) and KHB (right panel). **b** Normal livers (*n* = 9) perfused with KHB + 64 μM Meb (left panel) and KHB (right panel). **c** Cholestatic livers lacking Mrp2 (*n* = 6) perfused with KHB + 200 μM Bopta (left panel) and KHB (right panel). Bopta accumulation in normal liver (**a**) fitted a segmental linear regression, whilst MEB accumulation (**b**) fitted a continuous hinge function (gentle connections between the two lines). Both regressions are described by two lines *L*_1_ and *L*_2_ intersecting at *T*_0_ or time when bile excretion started to impact the accumulation. In livers lacking Mrp2, Bopta accumulation (**c**) fitted a single linear regression. During the rinse period, liver concentration decay fitted a one-phase decay for Bopta and Meb (**a**, **b**, right). No Bopta decay was observed in the absence of Mrp2 (**c**, right)

Bopta liver accumulation fitted a segmental linear regression, whilst Meb accumulation fitted a continuous hinge function (Fig. 3a, b, left panels). Both regressions were described by two lines *L*_1_ and *L*_2_ intersecting at *T*_0_ or time when bile excretion started to impact the accumulation. A gentle transition between the two lines characterised Meb (Fig. 3b, left panels, red fit), whilst Bopta transition was abrupt (Fig. 3a, left panel, red fit). *T*_0_ was slightly shorter for Meb (5.5 ± 1.1 min) than Bopta (8.0 ± 0.5 min) (*p* < 0.001, Table 2). The first lines (*L*_1_) below *T*_0_ had a slope of 37 ± 5 μM/min for Bopta and 333 ± 71 μM/min for Meb (*p* < 0.001). Thus, Meb entry into livers was much higher than Bopta entry. The second lines (*L*_2_ for time > *T*_0_) had lower slopes (8 ± 1 μM/min for Bopta and 16 ± 5 μM/min for Meb, *p* < 0.001). After *T*_0_, liver concentrations reflected the balance between uptake and excretory transporter activities. Bile concentrations were detectable 5 min after the perfusion start: 13,994 ± 2,391 μM for Meb and 2,107 ± 710 μM for Bopta (*p* < 0.001). During the rinse period, Bopta and Meb decay of liver concentrations fitted a one-phase decay (Fig. 3a, b, right panels) with a higher *k*_el,liver_ for Meb (0.13 ± 0.02 min^−1^) than for Bopta (0.09 ± 0.01, *p* < 0.001, Table 2).

**Table 2:** Bopta and Meb accumulation and decay parameters in livers and hepatocytes

Imaging agents	Bopta	Meb	*p*, Bopta ***versus*** Meb	Bopta
Livers	Normal	Normal		no Mrp2
*T*_0,liver_ [min]	8.0 ± 0.5	5.5 ± 1.1	< 0.001	-
*T*_0,HC_ [min]	4.7 ± 1.3	4.0 ± 1.5	.37	-
_Slope_*L*_1,liver_ [μM/min]	37 ± 5	333 ± 71	< 0.001	28 ± 6*
_Slope_*L*_1,HC_ [μM/min]	57 ± 16	452 ± 107	< 0.001	35 ± 8**
_Slope_*L*_2,liver_ [μM/min]	8 ± 1	16 ± 5	< 0.001	-
_Slope_*L*_2,HC_ [μM/min]	8 ± 2	22 ± 7	< 0.001	-
*k*_el,liver_ [min^−1^]	0.09 ± 0.01	0.13 ± 0.02	< 0.001	-
*k*_el,HC_ [min^−1^]	0.10 ± 0.02	0.16 ± 0.03	< 0.001	-
*T*_1/2,liver_ [min]	7.6 ± 0.9	5.5 ± 0.7	< 0.001	-
*T*_1/2,HC_ [min]	7.4 ± 1.3	4.5 ± 0.6	< 0.001	-
CL_el,HC_ [mL/min]	0.95 ± 0.17	1.44 ± 0.26	< 0.001	-

Bopta and Meb accumulation in normal livers and hepatocytes fitted nonlinear regressions described by two lines *L*_1_ and *L*_2_ intersecting at *T*_0_ or time when bile secretion started to impact the accumulation profile. During decay, liver and hepatocyte concentrations fitted a one-phase decay described by *k*_el_ and *T*_1/2_. CL_el,HC_ was calculated by *k*_el_ · *V*_HC_. Statistics between normal livers and livers lacking Mrp2 (column 2 *versus* column 5) are *p* < 0.010 (*) and *p* < 0.001 (**). *CL*_*el*,*HC*_ Elimination clearance from hepatocytes, *k*_*el*_ Elimination constant rate, *HC* Hepatocytes, *T*_*1/2*_ Half-life of elimination, *V*_*HC*_ Hepatocyte volume

In livers lacking Mrp2, Bopta accumulation fitted a single linear regression (*L*) during the entire perfusion period (Fig. 3c, left panel). *T*_0_ value was not available and Bopta bile excretion was minor. The *L* slope (28 ± 6 μM/min) was significantly lower than the *L*_1_ slope measured in normal livers (37 ± 5 μM/min, *p =* 0.01), showing a decreased Bopta entry in cholestatic livers. There was no decay during the rinse period and Bopta remained trapped inside hepatocytes.

### Accumulation and decay profiles of Bopta and Meb hepatocyte concentrations

All pharmacokinetic parameters were also measured in hepatocytes to eliminate the impact of the extracellular and canaliculi compartments on the results. In hepatocytes, both Bopta and Meb accumulation fitted a continuous hinge function during the perfusion period (data not shown). *T*_0_ were similar in both groups (Table 2). *L*_1_ and *L*_2_ slopes were significantly lower for Bopta than for Meb. During the decay period, the decline of hepatocyte concentrations fitted a one-phase decay with a *k*_el,HC_ higher for Meb than for Bopta (Table 2). The elimination clearance from hepatocytes (CL_el,HC_) was significantly higher for Meb than Bopta.

### Parameter comparisons between liver and hepatocytes

Despite small discrepancies, liver and hepatocyte parameters were close. Bopta and Meb *T*_0,liver_ was longer than the respective *T*_0,HC_ (Table 2). Bopta and Meb *L*_1,liver_ slopes were lower than *L*_1,HC_ slopes. *L*_2,liver_ and *L*_2,HC_ slopes were identical for both agents. Bopta *k*_el_ were similar in hepatocytes and livers. Meb *k*_el_ was slightly higher in hepatocytes than in livers.

### Effect of acquisition frequency on pharmacokinetic parameters

**Fig. 4:**
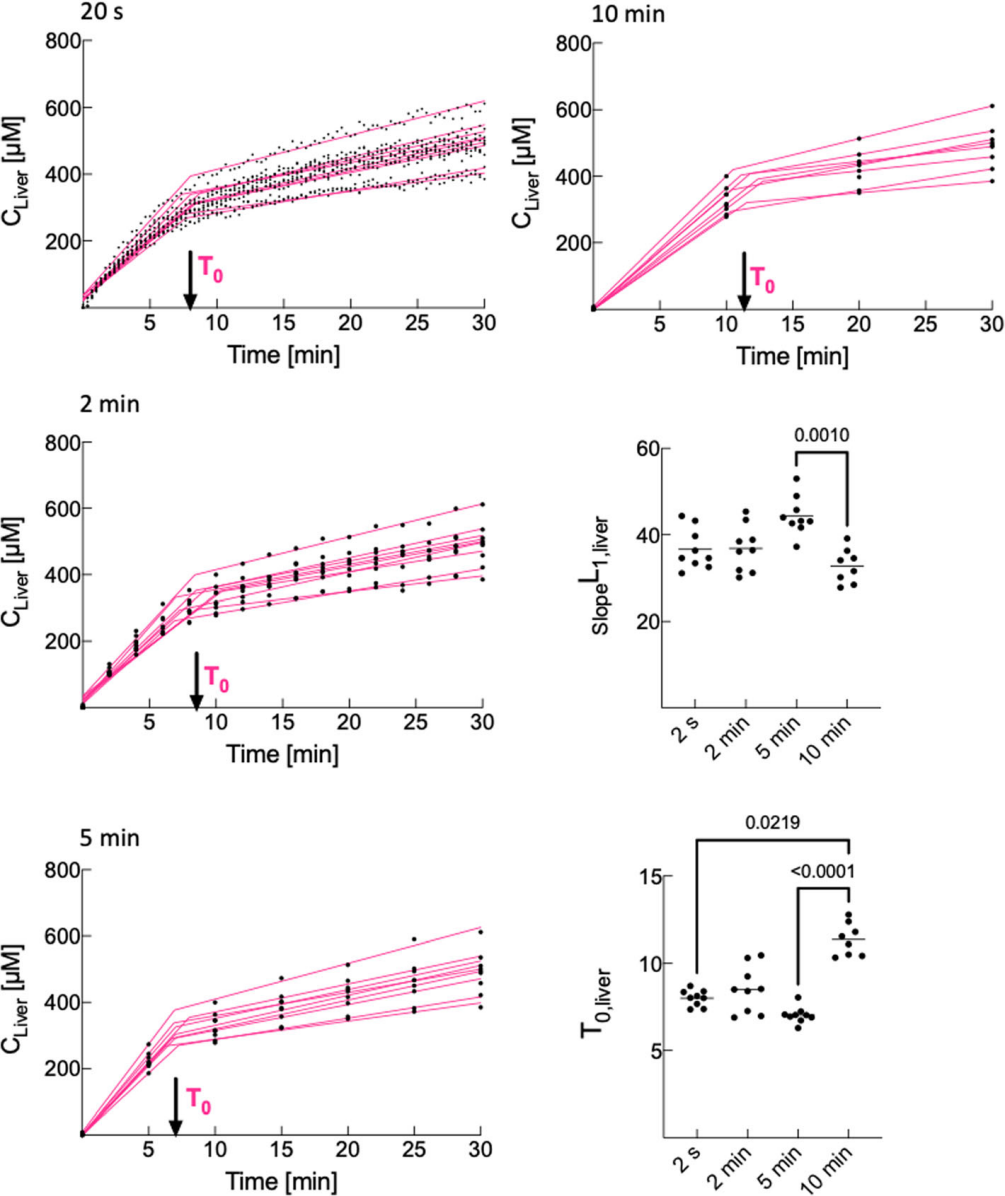
Effect of acquisition frequency on pharmacokinetic parameters. We simulated lower acquisition frequencies and fitted Bopta liver concentrations with a segmental linear regression. Similar *T*_0_ and *L*_1_ slopes were obtained with acquisition frequency between 20 s and 5 min

To allow more easy translation to clinical imaging, we determined the lower acquisition frequency that gives pharmacokinetic parameters similar to those measured with a 20-s frequency (Fig. 4). Similar Bopta and Meb *T*_0_ and *L*_1_ slopes were obtained with acquisition frequencies of 20 s, 2 min, and 5 min. Bopta and Meb decay parameters were similar from 20 s to 10 min (data not shown).

## Discussion

Our study describes how liver extraction ratios and cholestasis interfere with the compartmental distribution of hepatobiliary agents. Meb provides much higher maximal liver concentrations than Bopta, with hepatocytes contributing to 78% of liver concentrations [20]. Meb in the extracellular compartment does not contribute much to liver concentrations, whilst Bopta in the extracellular compartment participates to liver concentrations all along the accumulation period. The contribution of bile canaliculi to liver concentrations (close to 16%) is similar for both agents. Thus, Meb liver concentrations mainly rely on hepatocytes and bile canaliculi, whereas Bopta liver concentrations also depend on the extracellular compartment. In comparison to Bopta, Meb chemical structure has a huge affinity for the sinusoidal transporter Oatp. The second hepatobiliary MR agent gadoxetic acid which is widely used in clinical liver imaging has a higher liver extraction ratio than Bopta and its liver pharmacokinetics should be intermediate between Meb and Bopta [23–25].

In livers lacking Mrp2 and perfused with Bopta, liver concentrations only rely on the hepatocyte and extracellular compartments. The low Bopta concentrations in bile canaliculi were associated with higher hepatocyte concentrations in comparison to normal livers. This increased hepatocyte accumulation does not depend on Bopta hepatocyte uptake which is decreased. Indeed, the *L* slope during Bopta accumulation measured in livers lacking Mrp2 was lower that the *L*_1_ slope of normal livers. This result points out that high signal enhancements are not necessarily associated with high hepatocyte uptake. During the rinse period, Bopta remained trapped inside hepatocytes. Such results were published previously *in vivo* when gadoxetic acid was injected in rodents lacking Mrp2 [26].

Besides Bopta and Meb compartmental distribution, the model identifies several pharmacokinetic parameters obtained by analysing the accumulation of liver or hepatocyte concentrations. During accumulation, the time when agent excretion (in bile canaliculi or back to sinusoids) impacts hepatocytes and liver concentrations (*T*_0_) is important. *T*_0_ is lower than 10 min and close for Bopta and Meb in normal livers. Before *T*_0_, *L*_1_ slope characterises Bopta and Meb entry (in μM/min) into hepatocytes or livers. The value is much higher for Meb than Bopta. After *T*_0_, the accumulation rates decrease and *L*_2_ slopes are much lower than *L*_1_ slopes for both agents. Concomitant entry and efflux from hepatocytes or livers explain the lower *L*_2_ slopes. Similar biphasic accumulations were previously published following bolus injection of imaging agents in patients [4, 27–29]. In these studies, slopes and *T*_0_ were not measured by nonlinear regressions. In our study, we used two linear regressions provided by the software GraphPad Prism [21, 22]. Saito et al. [30] also showed a biphasic liver accumulation in patients with or without cirrhosis following a bolus injection of gadoxetic acid. Interestingly, the accumulation rates in the second phase (similar to our *L*_2_ slope) decreased with the severity of cirrhosis.

In clinical practice, the hepatobiliary phase is obtained 20 min (gadoxetic acid) or later (Bopta) after injection. These time-points are superior to *T*_0_ and are situated in the second accumulation phase when liver concentrations depend on both uptake and efflux transporters. Consequently, the clinical time-points underestimate the entry of agents into hepatocytes. Another evidence of early agent excretion from hepatocytes is provided by bile concentrations. For both agents, bile concentrations were higher than 2,000 μM 5 min after the perfusion start. In clinical practice, it is not possible to acquire images every 20 s as done in our study. With simulations, we show that the values of *T*_0_ and *L*_1_ slope are similar until a 5-min acquisition frequency. Another important information for human imaging is that despite discrepancies, liver and hepatocyte parameters of pharmacokinetics were close.

The main pharmacokinetic parameter obtained by analysing the decay of Bopta and Meb concentrations was the elimination clearance from hepatocytes. Meb CL_el,HC_ was higher than Bopta CL_el,HC_ and null in cholestatic livers. However, this parameter does not differentiate bile efflux from efflux back into sinusoids. The distinction is unimportant for Meb which is not transported by Mrp3 back into sinusoids. In contrast, Bopta can efflux back to sinusoids [15, 19].

The isolated and perfused rat liver is an *ex vivo* model that allows the mechanistic distribution of agents and tracers in liver compartments. By placing a gamma counter over livers, we can mimic imaging systems such as magnetic resonance imaging (MRI) and single-photon computed emission tomography (SPECT). We can calculate concentrations in all liver compartments. The model clearly distinguishes *in situ* concentrations from concentrations detected by the counter, knowing that hepatocyte membrane transporters govern *in situ* concentrations. Liver concentrations detected by the counter is the sum of three *in situ* concentrations corrected by their respective volume ratio to liver [11]. An important restriction is that a unique volume of interest is investigated that must be delineated in homogenous tissues. The model can be applied to livers with cirrhosis and steatosis [31, 32].

The isolated and perfused rat liver is a convenient model because the experimental conditions are well controlled and simplified. It was useful to determine Bopta and Meb compartmental distribution, an issue inaccessible in *in vivo* imaging. The model is frequently used to investigate the pharmacokinetics of drugs, liver concentrations being measured by collecting biopsies over time. However, this procedure deteriorates the preparation. Such deterioration is avoided by placing a gamma counter over livers. Other experimental models, such as cultured cells or 3D tissues are not appropriate because they do not preserve the liver architecture and liver perfusion flow.

In our model, livers are perfused only through the portal vein, a condition that changes the physiological dual-input liver perfusion. However, hepatic arterial flow represents only 25% of liver blood flow. Moreover, the low portal vein pressure (10–12 mmHg) remained steady during the entire protocol. There is no recirculation of solutions which are discarded after the first pass through sinusoids. This approach is useful to measure the pharmacokinetic parameters we need such as the liver extraction ratio (*C*_in_ − *C*_out_)/*C*_in_. Bopta and Meb are not metabolised and the count rates we measure only include parent compounds. We perfuse steady concentrations of agents during 30 min, whilst Meb and Bopta are injected in clinical protocols. Following Bopta injection, human plasma concentrations remain high because the liver extraction ratio is low and Bopta remains in the systemic circulation. In contrast, Meb is rapidly cleared from the systemic circulation in patients and the perfusion we used does not mimic the clinical situation.

In conclusion, our model describes how liver extraction ratios and cholestasis interfere with the compartmental distribution of hepatobiliary agents and reveals new parameters that characterise the accumulation and decay of Bopta and Meb liver concentrations.

## Data Availability

Data are presented in the main manuscript.

## Abbreviations

Bopta: Gadobenate dimeglumine
*C*_bile_ [μM]: Concentration in bile duct and canaliculi
*C*_BC_ [μM]: Concentration in bile canaliculi measured by the counter
*C*_EC_ [μM]: Extracellular concentration
*C*_HC100% _ [μM]: *In situ* hepatocyte concentration
*C*_HC78%_ [μM]: Concentration in hepatocytes measured by the counter
*C*_in_ [μM]: Perfused concentration in portal vein
CL_el_ [mL/min]: Elimination clearance from hepatocytes
*C*_liver_ [μM]: Liver concentration
*C*_out_ [μM]: Concentration in hepatic veins
Dtpa: Gadopentetate dimeglumine
*k*_el_ [min^−1^]: Decay (elimination) rate constant
KHB: Krebs-Henseleit-Bicarbonate
Meb: Mebrofenin
MRI: Magnetic resonance imaging
Mrp2: Multidrug resistance associated protein 2
Mrp3: Multidrug resistance associated protein 3
Oatp: Organic anion transporting polypeptide
*Q*_bile _ [μL/min/liver]: Bile flow rate
*Q*_H _ [mL/min]: Liver flow rate
_slope_*L*_1_: Early accumulation of liver and hepatocyte concentrations
_slope_*L*_2_: Late accumulation of liver and hepatocyte concentrations
*T*_0 _ [min]: Time when bile excretion impacts hepatocyte and liver accumulation
*V*_HC_ [mL]: Hepatocyte volume
